# Legal Medicine, Vol. II

**Published:** 1884-11

**Authors:** 


Legal Medicine, Vol. II. *Legitimacy, Abortion, Rape, Sodomy, Infanticide, Drowning, Hanging, Suffocation. By* Ch. Meymott Tidy. *Royal, 8vo., sheep, pp. xxiv., 508. Philadelphia:* Henry C. Lea's Son & Co. 1884.

We have had the pleasure of reviewing in the pages of the Journal the first volume of this work, as published in two separate volumes, by the firm of Wood & Co., of New York, and we must say that the firm of Lea, in issuing such a beautiful edition, does credit to the value of this admirable work. A first glance at such a store of varied information, so rapidly issued, reminds one of Washington Irving's "The Art of Book Making," but the simile ends with this suggestion, for the wealth of information is probably surpassed by the interest of the style and the judgment of the author.

We cannot say more than what we expressed in the JOURNAL of October, 1883, regarding this work of Medical Jurisprudence. We think that it is supplying the want of many, and that it is fully up to the times, as far as English Law is concerned. H. D. V.

